# Organ Malperfusion and Mortality in Acute Type 1 Aortic Dissection with Iliac Extension: A Retrospective Single-Center Study Focused on Mesenteric Malperfusion and the Source–Clearance Hypothesis

**DOI:** 10.3390/jcm15207958

**Published:** 2026-10-14

**Authors:** Ali Onder Kilic

**Affiliations:** Department of Cardiovascular Surgery, Bursa City Hospital, Bursa Faculty of Medicine, University of Health Sciences, 16110 Bursa, Türkiye; draok24@gmail.com

**Keywords:** aortic dissection, malperfusion, mesenteric ischemia, lactate-to-albumin ratio, mortality

## Abstract

**Background/Objectives:** Mesenteric malperfusion is the most lethal and least recognizable complication of acute type A aortic dissection. This study aimed to examine organ-specific malperfusion patterns and 30-day mortality in acute type 1 aortic dissection with iliac extension, and to evaluate whether the lactate-to-albumin ratio (LAR) and superior mesenteric artery (SMA) origin pattern add predictive value for mesenteric malperfusion syndrome within a “source–clearance” framework. **Methods:** In this retrospective single-center study, 80 consecutive patients with acute type 1 aortic dissection extending to the iliac arteries who underwent surgical repair between January 2020 and March 2026 were reviewed. Malperfusion (preoperative finding) and malperfusion syndrome (end-organ injury persisting despite central repair) were defined for six organ systems; visceral branch origin and planimetric true lumen ratio were assessed on computed tomography angiography (CTA). The primary endpoint was 30-day mortality. **Results:** Thirty-day mortality was 33.8%; 60% of patients had malperfusion, and mortality rose with the number of organs involved (0/1/≥2: 12.5%/32.1%/70.0%). Mesenteric malperfusion (*n* = 21) carried the highest mortality (71.4%); all nine patients who developed mesenteric syndrome died. Cerebral malperfusion was most frequent (*n* = 27); all nine who improved postoperatively survived, whereas mortality was 81.8% in the 22 who developed the syndrome. Preoperative shock or cardiopulmonary resuscitation (CPR) (odds ratio [OR] = 7.02), mesenteric malperfusion (OR = 6.24), LAR (OR = 1.07 per 0.01 unit) and Glasgow Coma Scale (GCS; OR = 0.75 per point) were independent predictors. LAR and SMA origin pattern were uncorrelated (r = −0.04) and together raised the area under the receiver operating characteristic curve (AUC) for mesenteric syndrome from 0.861 to 0.955; mortality was 75% with mixed SMA origin (*n* = 8). **Conclusions:** Malperfusion is a dose-dependent risk spectrum; mesenteric involvement is its most lethal component, increasing mortality six-fold independently of hemodynamic and metabolic status. Joint interpretation of LAR and SMA origin pattern offers a practical, validation-pending framework for perioperative management of the mesenteric circulation in centers without a hybrid operating room.

## 1. Introduction

Acute type A aortic dissection is a cardiovascular emergency with a reported incidence of 2.5–6 per 100,000 person-years, rising to 8.7 per 100,000 in recent population-based data as the population ages; patients older than 75 years are projected to account for more than half of all dissections by 2050 [1,2]. The early natural history remains unforgiving: untreated, mortality increases by 1–2% per hour and reaches 30–68% within two days [1]; in the International Registry of Acute Aortic Dissection (IRAD), 48 h mortality was 23.7% under medical management versus 4.4% in patients treated surgically [3], and in-hospital mortality after surgical repair has declined from about 25% to 18% over two decades [4,5]. Early (30-day or in-hospital) mortality after surgery is uniformly around 17% in multicenter registries (GERAADA 17%, IRAD 18%, NORCAAD 16%) [1,6] and in the same range in the European (ERTAAD) and UK national series [7,8], whereas the US Society of Thoracic Surgeons database reports operative mortality of 27% [9]; in high-risk subgroups—shock, tamponade, malperfusion—mortality exceeds 40% [6,10]. These figures refer to all-comer Stanford type A populations, in which dissections confined to the ascending aorta (DeBakey type II) and those propagating to the iliac arteries are pooled; no registry reports outcomes specifically for DeBakey type I dissection with iliac extension, and the extent of distal propagation is captured only indirectly, through malperfusion. Because the Stanford type A designation also encompasses DeBakey type II dissections, we use the term “type 1 aortic dissection with iliac extension” for a population in which visceral and iliac malperfusion can occur.

Malperfusion denotes insufficient perfusion of an end organ caused by dissection-related obstruction of its supplying branch; roughly four in five cases are dynamic (flap-dependent), the remainder static [11], and the term malperfusion syndrome is reserved for the stage at which tissue necrosis or organ dysfunction has already developed [12]. Among all types, mesenteric involvement carries the highest adjusted odds of in-hospital death in IRAD (adjusted OR = 4.84) [1]; it is also the hardest to recognize, because its clinical course is frequently silent and no circulating marker specific to intestinal ischemia exists. Lactate is usually interpreted as a marker of mesenteric ischemia [13,14,15]; however, its level reflects not only production but also clearance capacity. In this study, we propose a model that treats circulating lactate as the net result of production (source) and elimination (clearance), and use the lactate-to-albumin ratio (LAR) as a single-number marker of this balance.

The aims of this study were, in acute type 1 aortic dissection with iliac extension, (i) to examine the relationship between organ-specific malperfusion and malperfusion syndrome patterns and 30-day mortality; (ii) to build on planimetric/anatomical assessment approaches [16,17,18] to evaluate the complementary value of branch-origin pattern, true lumen ratio and LAR in predicting mesenteric malperfusion syndrome; and (iii) to propose a practicable perioperative decision framework for centers without a hybrid operating room.

## 2. Materials and Methods

### 2.1. Study Design and Population

**Figure 1:**
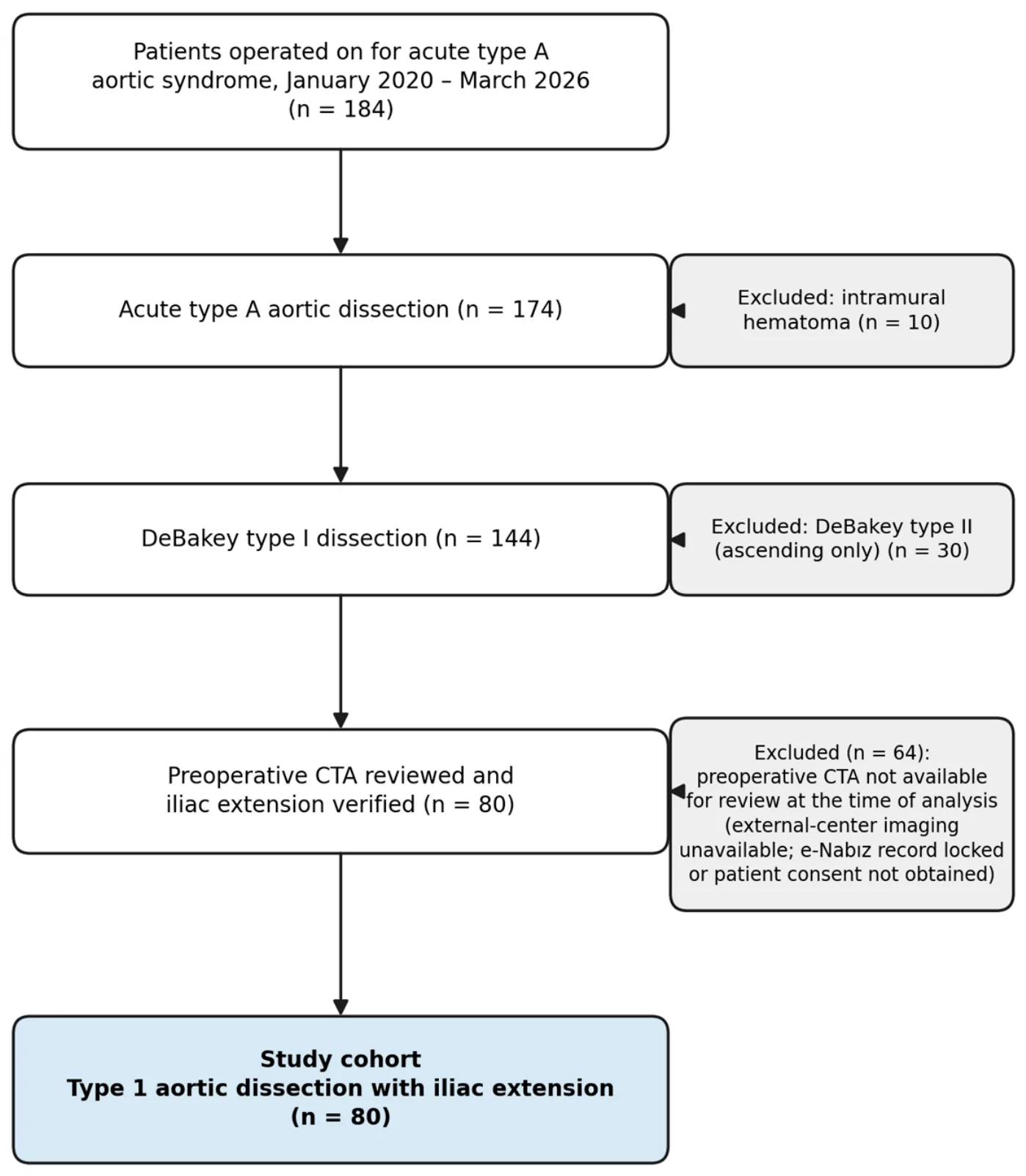
Flow chart of the study population. IMH: intramural hematoma; CTA: computed tomography angiography.

This retrospective single-center study included patients operated on for acute type 1 aortic dissection between January 2020 and March 2026; it was approved by the Bursa City Hospital Ethics Committee (2026-08/15), and the requirement for informed consent was waived owing to the retrospective design. Inclusion criteria were: (1) acute type 1 aortic dissection; (2) technically completed surgical repair at our center; (3) preoperative computed tomography angiography (CTA) confirming extension to the iliac arteries; and (4) available preoperative clinical and laboratory data. Of 184 patients operated on for acute type A aortic syndrome in this period, 10 had intramural hematoma and 30 had DeBakey type II dissection; of the remaining 144 patients with type I dissection, 64 were excluded because preoperative CTA was not available for review at the time of analysis (external-center imaging unavailable, national e-Nabız records locked or patient consent not obtained), leaving 80 patients in the cohort (Figure 1).

Thirteen patients died within the first 24 postoperative hours (5 in the operating room); no patient was excluded, to avoid selection bias. Preoperative variables were complete; because postoperative missing data (9 of 27 deaths) resulted from early death (missing not at random, MNAR), complete-case analysis was preferred.

### 2.2. Data Collection, Imaging and Laboratory

Clinical, laboratory and radiological data were obtained from the hospital information system and the national teleradiology database. Demographic and clinical data (age, sex, body mass index (BMI), comorbidities, preoperative Glasgow Coma Scale [GCS], history of cardiopulmonary resuscitation [CPR], shock, ejection fraction, grade of aortic regurgitation [European Society of Cardiology (ESC) 2021], pericardial effusion/tamponade) and laboratory parameters (complete blood count, creatinine, albumin, alanine aminotransferase (ALT), aspartate aminotransferase (AST), lactate, troponin, C-reactive protein (CRP), D-dimer) were collected. **Lactate and LAR protocol.** Lactate was measured on arterial blood gas samples (ABL90 FLEX; Radiometer Medical ApS, Brønshøj, Denmark) and albumin on the simultaneous venous biochemistry sample. LAR was calculated as lactate (mmol/L)/albumin (g/L) at three predefined time points: (i) preoperative—the arterial blood gas drawn from the arterial line immediately before induction of anesthesia (an arterial line was placed on arrival in every patient, including those under CPR, and the first pre-bypass arterial sample was used); (ii) post-bypass—the first arterial blood gas after separation from cardiopulmonary bypass and protamine administration; and (iii) intensive care unit (ICU)—the first arterial blood gas on ICU admission. Only the preoperative value entered the predictive analyses; the post-bypass and ICU values were used descriptively. Lactate from the venous line of the bypass circuit, which is discussed as a recommendation in Section 4.7, was not systematically recorded in this cohort. The true lumen ratio (TLR) was measured planimetrically at the celiac, superior mesenteric (SMA) and renal artery levels as the ratio of true-lumen area to total luminal area on the arterial phase of single-phase CTA; the origin of each branch was classified as true (T), false (F) or mixed (T/F; ostium supplied from both lumens), and the adjusted true lumen ratio (TLAR; %TLR for true origin, 100−%TLR for false origin) was defined. The obstruction mechanism was classified as static when intra-branch dissection or thrombosis was visible and as dynamic when the ostium was patent but compressed by the flap; because single-phase CTA cannot show flap motion, this classification is necessarily approximate (see Section 4.7 and Section 4.8). TLR was not measured at the arch level, where flap pulsatility precludes a reliable contour on single-phase CT; arch branches were assessed by dissection/thrombosis pattern. All measurements were performed by a single experienced surgeon using OsiriX MD (Pixmeo SARL, Bernex, Switzerland) and Horos, version 4.0 (Horos Project, open-source).

### 2.3. Definitions of Malperfusion

Malperfusion (preoperative anatomical and/or clinical finding) and malperfusion syndrome (end-organ injury persisting despite central repair) were defined separately for each organ. **Coronary:** preoperative troponin > 150 ng/L (independence from tamponade verified by comparing troponin according to coronary involvement in patients without tamponade: median 209.1 vs. 12.8 ng/L) and/or intraoperative confirmation. Syndrome: postoperative myocardial infarction, persistent low output, or need for percutaneous coronary intervention (PCI)/coronary artery bypass grafting (CABG). **Cerebral:** carotid/innominate dissection or thrombosis on CTA and/or preoperative GCS < 13 or focal neurological deficit. Syndrome: persistent deficit/stroke/coma. For the GCS trajectory analysis (Section 3.3), “good” neurological status was defined as GCS ≥ 13 and “poor” as GCS < 13, both preoperatively (last assessment before induction; in intubated patients the documented pre-intubation GCS when available; otherwise the GCS assessed by our team on arrival, with the verbal component recorded as not testable and the total taken as E + M, so that such patients scored ≤10 and were classified as poor preoperative status) and postoperatively (best GCS recorded during the ICU stay after withdrawal of sedation); patients who died in the operating room or before awakening were classified as poor postoperative status. **Mesenteric:** celiac/SMA thrombosis or dissection on CTA, or TLAR < 25% combined with preoperative lactate >2.5 mmol/L, or clinical signs of mesenteric ischemia. Syndrome: bowel ischemia requiring laparotomy/endovascular intervention and resection. Because TLAR and preoperative lactate are components of the definition, they were not tested as predictors of mesenteric malperfusion; LAR, which is not part of the definition, was evaluated independently. **Renal:** false-lumen origin and/or oliguria, anuria, hematuria or flank pain; owing to bilateral redundancy, anatomical origin alone was not a criterion. Syndrome: Kidney Disease: Improving Global Outcomes (KDIGO) stage increase and/or renal replacement therapy. **Iliac:** iliac thrombosis on CTA or clinical limb ischemia with loss of femoral pulse. Syndrome: need for additional bypass. **Spinal:** postoperative paraplegia/paraparesis. Syndrome: need for cerebrospinal fluid (CSF) drainage.

### 2.4. Surgical Procedures, Follow-Up and Endpoints

The surgical strategy was determined by tear location and arch involvement as isolated ascending, Bentall, Wheat, valve-sparing, hemiarch or total arch replacement (± frozen elephant trunk, FET); cannulation was via the right axillary artery as standard and the femoral artery in instability/CPR. Arterial cannulation was via the right axillary artery in 71 patients (88.8%), femoral with conversion to axillary in 6, and directly femoral under CPR in 3. Venous cannulation was two-stage right atrial in 77 patients and femoral venous in 3 (previous cardiac surgery). Postoperative extracorporeal membrane oxygenation (ECMO) was instituted exclusively as central (ascending aortic graft/right atrial) veno-arterial postcardiotomy support for failure to wean from cardiopulmonary bypass despite repeated weaning attempts under maximal inotropic and vasopressor support; no patient received ECMO as a bridge to surgery or for isolated respiratory failure. The primary endpoint was 30-day mortality; secondary endpoints were organ-specific syndrome, acute kidney injury (AKI), neurological complications and need for ECMO.

### 2.5. Statistical Analysis

Analyses were performed with IBM SPSS Statistics, version 29.0 (IBM Corp., Armonk, NY, USA) and JASP, version 0.98.1 (JASP Team, University of Amsterdam, the Netherlands); continuous variables are presented as mean ± standard deviation (SD) or median (interquartile range, IQR), categorical variables as *n* (%), and compared with t-test/Mann–Whitney U and chi-square/Fisher tests. Demographic, laboratory, radiological (organ-specific malperfusion, branch origin, TLR/TLAR, CT-to-bypass time) and operative variables were screened in univariable analysis (Supplementary Tables S1–S3); preoperative variables with *p* < 0.10 in univariable analysis entered backward-elimination (*p* < 0.10) logistic regression, with collinear variables removed. The contribution of LAR and SMA origin pattern to discriminating mesenteric syndrome was examined by receiver operating characteristic (ROC) analysis after sequential and combined addition to a base model of age and hemodynamic status. Internal validation of the multivariable models was performed with 1000 bootstrap resamples; optimism-corrected AUCs were derived by Harrell’s method and 95% confidence intervals (CI) of the apparent AUC by the percentile method. *p* < 0.05 was considered significant for all tests; the study is reported in accordance with the Strengthening the Reporting of Observational Studies in Epidemiology (STROBE) statement.

### 2.6. Use of Generative Artificial Intelligence

During the preparation of this manuscript, the author used Claude (Anthropic, San Francisco, CA, USA; claude.ai, 2025–2026 versions) for language editing and Turkish-to-English translation. No generative artificial intelligence tool was used for data collection, statistical analysis or interpretation of the results. The author has reviewed and edited the output and takes full responsibility for the content of this publication.

## 3. Results

Thirty-day mortality was 33.8% (27/80) and in-hospital mortality 36.3%. Baseline, laboratory and malperfusion findings are shown in Table 1 (full list: Supplementary Tables S1 and S2). Non-survivors had lower GCS, more frequent tamponade and instability, higher lactate, LAR and creatinine, and lower albumin and platelets; CT-to-bypass time was paradoxically shorter (192 vs. 241 min; *p* = 0.034). Mortality by procedure: isolated ascending replacement 5/20, Bentall 5/16, Wheat 3/7, hemiarch 5/18, total arch 9/25, total arch + FET 4/4 (Fisher *p* = 0.011), valve-sparing 1/2; procedures were combined in 12 patients, so these denominators sum to more than 80. Mortality did not differ between arch-extending procedures (*n* = 47) and ascending/root procedures (*n* = 33) (38.3% vs. 27.3%; *p* = 0.34). With concomitant CABG (*n* = 8), mortality was 62.5% vs. 30.6% (*p* = 0.11). Cardiopulmonary bypass (154 vs. 260 min; *p* < 0.001) and cross-clamp time (92 vs. 136 min; *p* = 0.002) were associated with mortality, whereas antegrade cerebral perfusion (*p* = 0.11) and total circulatory arrest (*p* = 0.24) times were not. Four patients (5.0%) required central veno-arterial postcardiotomy ECMO for failure to wean from bypass; three of them had presented in shock or under CPR, three had mesenteric malperfusion, post-bypass lactate ranged from 14.4 to 26.0 mmol/L, and all four died within 48 h of surgery. ECMO in this cohort therefore marks the end-stage of combined cardiogenic and metabolic failure rather than an independent treatment strategy.

### 3.1. Independent Predictors of Mortality

In univariable analysis, shock/CPR, mesenteric and cerebral malperfusion, tamponade, creatinine, thrombocytopenia, lactate, LAR and GCS were significantly associated with mortality, and coronary malperfusion showed a trend (*p* = 0.06) (Supplementary Table S3). In the multivariable model, shock/CPR, mesenteric malperfusion, LAR and GCS remained independent (Table 2); as a single preoperative variable, LAR (AUC 0.745) discriminated better than lactate (AUC 0.715) or albumin (AUC 0.680). TLR values were not associated with mortality.

### 3.2. Organ-Specific Malperfusion and Syndrome

**Figure 2:**
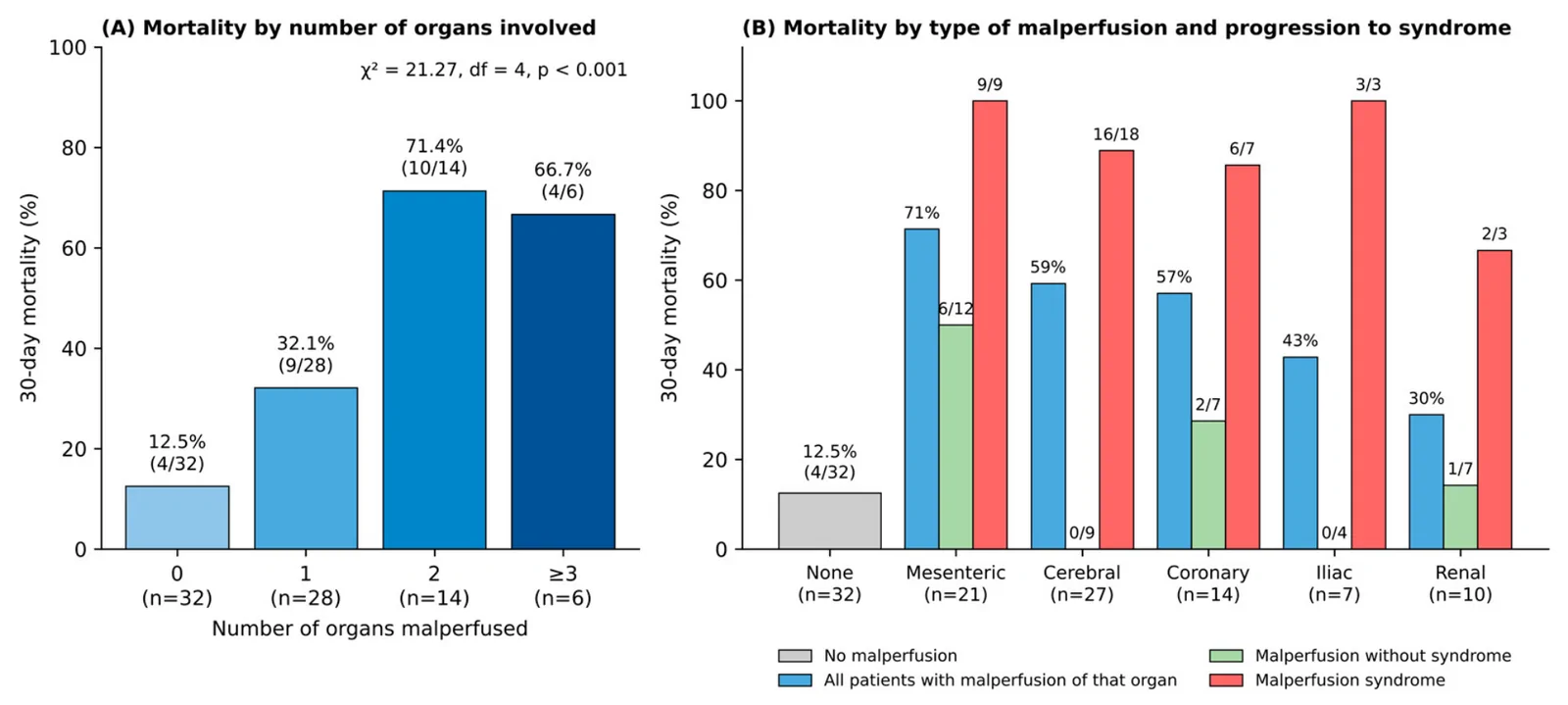
Thirty-day mortality (**A**) by number of organs malperfused and (**B**) by type of malperfusion, stratified by progression to malperfusion syndrome. Numbers above bars are deaths/patients. Syndrome bars include only patients with a preoperative malperfusion finding for that organ (Table 3).

Malperfusion was present in 60% of patients, and mortality increased with the number of organs involved (Table 3; Figure 2A). Mesenteric malperfusion showed the strongest association (univariable OR = 9.79, 95% CI 3.13–30.60; multivariable OR = 6.24; Table 2 and Table 3); all nine patients who developed mesenteric syndrome died. The malperfusion-to-syndrome gradient pointed in the same direction for every organ (Figure 2B); renal and iliac malperfusion were not associated with mortality. Acute kidney injury developed in 52 of 72 patients (72.2%) with evaluable postoperative creatinine (KDIGO stage 1/2/3: 24/16/12); mortality was 32.7% with AKI versus 10.0% without (*p* = 0.073), and six of eight patients who received renal replacement therapy died. Because this analysis excludes eight patients who died before a postoperative sample could be obtained, it underestimates the AKI–mortality relationship.

### 3.3. Cerebral Malperfusion and Postoperative Neurological Course

Cerebral malperfusion was present in 27 patients (33.8%). In 25 of these, the criterion was anatomical—dissection or thrombosis of the innominate or carotid arteries on CTA—and in 17 of the 27 preoperative GCS was <13; only 2 patients met the definition by GCS <13 alone without arch-branch involvement. Two patients presented with focal cerebral signs—one with left hemiplegia and conjugate gaze deviation in the setting of right carotid thrombosis (GCS 10), one comatose with gaze deviation and anisocoria (GCS 3)—and both died. In the remaining 25, the picture was altered consciousness without lateralizing signs, typically in the setting of shock/tamponade/arrest. Limb weakness attributable to iliac or spinal malperfusion (two patients) was considered peripheral and was not classified as a cerebral deficit. Mortality was 60.0% (15/25) in patients with CT-documented arch-branch involvement and 50.0% (1/2) in the two patients identified by GCS alone. The postoperative course was decisive: of the 27 patients, 9 improved postoperatively and all survived, whereas 16 of the 18 who developed the syndrome died (88.9%); when the 4 patients who developed cerebral syndrome without a preoperative finding are included, syndrome mortality was 18/22 (81.8%; *p* < 0.0001). By GCS trajectory (good: GCS ≥ 13; poor: GCS < 13), mortality was 2.2% (1/45) in the good-preoperative/good-postoperative group, 0% (0/4) in poor/good, 77.8% (14/18) in good/poor and 92.3% (12/13) in poor/poor; the five patients who died on the table were classified as “poor postoperative.” In the good/poor group, cardiopulmonary bypass (236 vs. 152 min; *p* = 0.009), cross-clamp (135 vs. 91 min; *p* = 0.019) and axillary cannulation times (41 vs. 23 min; *p* = 0.038) were significantly longer.

### 3.4. Preoperative Predictors of Mesenteric Malperfusion

**Figure 3:**
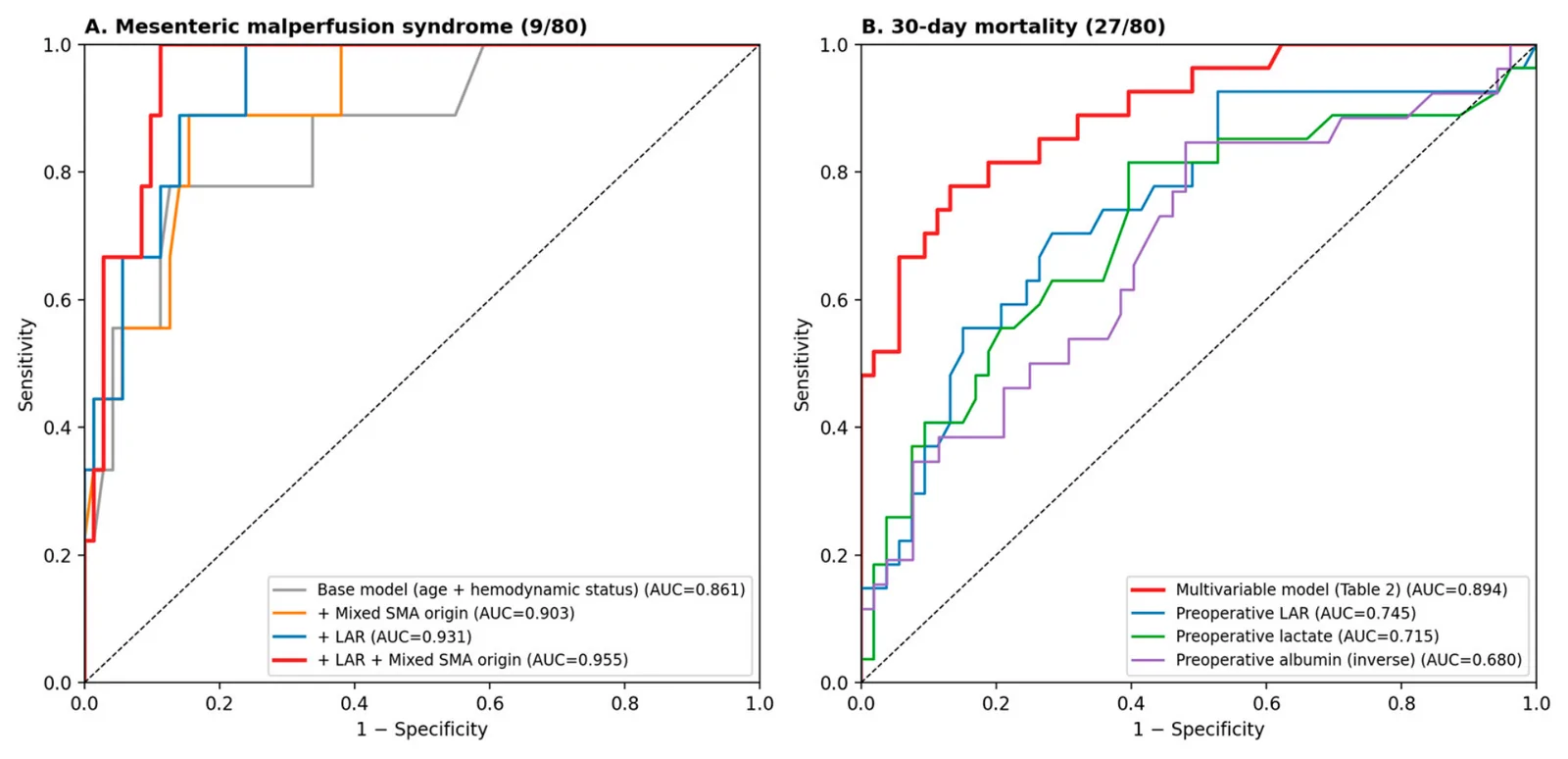
ROC curves. (**A**) Increase in discrimination for mesenteric malperfusion syndrome when mixed SMA origin, LAR, and both are added to the base model. (**B**) Multivariable model for 30-day mortality (Table 2) and preoperative LAR, lactate and albumin alone. All values are apparent (internal) AUCs.

The SMA more often originated from the false or a mixed lumen in non-survivors (*p* = 0.03). Mortality was 75.0% in the eight patients with mixed (T/F) SMA origin (pure true 27.1%, pure false 38.5%; *p* = 0.016; OR = 7.29; 95% CI 1.36–39.06); this subgroup did not differ from the rest of the cohort in age, GCS, creatinine or LAR, whereas hemodynamic instability (shock or CPR) was more frequent (4/8 [50.0%] vs. 14/72 [19.4%]). Preoperative LAR tended to be higher in patients with mesenteric malperfusion (median 0.118 vs. 0.067; *p* = 0.056) and was uncorrelated with SMA origin pattern (r = −0.04). Because the mesenteric definition incorporates lactate and SMA involvement, discrimination was analyzed against mesenteric syndrome, an endpoint independent of the definition: the base model had an AUC of 0.861, rising to 0.903 with mixed origin, 0.931 with LAR, and 0.955 with both (Figure 3A). Given the risk of overfitting with nine events, bootstrap internal validation (1000 resamples) was applied: the apparent AUC of the four-variable model was 0.955 (bootstrap percentile 95% CI 0.914–1.000) and the optimism-corrected AUC 0.909, versus 0.861 (95% CI 0.718–0.979) and 0.847 for the base model, so the increment was preserved on internal validation. D-dimer was not directly associated with mortality (*p* = 0.09; AUC = 0.619) but correlated with preoperative lactate and LAR (Spearman ρ = 0.30–0.32; *p* < 0.01) and inversely with platelet count (ρ = −0.22; *p* = 0.056). Platelet count was associated with mortality (median 160 vs. 197 × 10^3^/µL; *p* = 0.015), and thrombocytopenia (<150 × 10^3^/µL; *n* = 20) increased mortality risk (Fisher *p* = 0.029; OR = 3.36).

### 3.5. Summary of Principal Findings

In this cohort of 80 patients with acute type 1 aortic dissection extending to the iliac arteries, 30-day mortality was 33.8% and rose stepwise with the number of malperfused organs. Mesenteric malperfusion was the single strongest organ-specific predictor and, together with preoperative shock/CPR, LAR and GCS, remained independent in the multivariable model (Figure 3B); none of the nine patients who progressed to mesenteric syndrome survived. Preoperative LAR and mixed SMA origin carried non-overlapping information, and their combination improved discrimination of mesenteric syndrome on internal validation. The postoperative neurological course, rather than the preoperative finding, determined outcome in cerebral malperfusion.

## 4. Discussion

### 4.1. Anatomical Extension and Organ Malperfusion

Despite advances in surgical technique, operative mortality in acute type A aortic dissection remains high [1,2,19,20]; the principal determinant is the patient’s preoperative condition rather than the operation itself—rupture, tamponade, shock and, above all, malperfusion [11]. In our cohort, mesenteric malperfusion increased mortality six-fold independently of shock, GCS and LAR. Malperfusion is a direct consequence of anatomical extension: as false-lumen flow propagates the flap distally, it may create dynamic or static obstruction at each branch ostium [11,16]; a proximal tear increases false-lumen pressure and extension, and in IRAD, patients with mesenteric malperfusion more often had a flap originating at the aortic root (62.7% vs. 45.2%) and more frequent arch-branch and renal involvement [21]. Restricting the cohort to dissections with iliac extension produced a population in which all visceral beds were exposed to this mechanism; our malperfusion prevalence (60%) exceeding the 16–42% range in the literature [6,9] is consistent with that selection.

**Figure 4:**
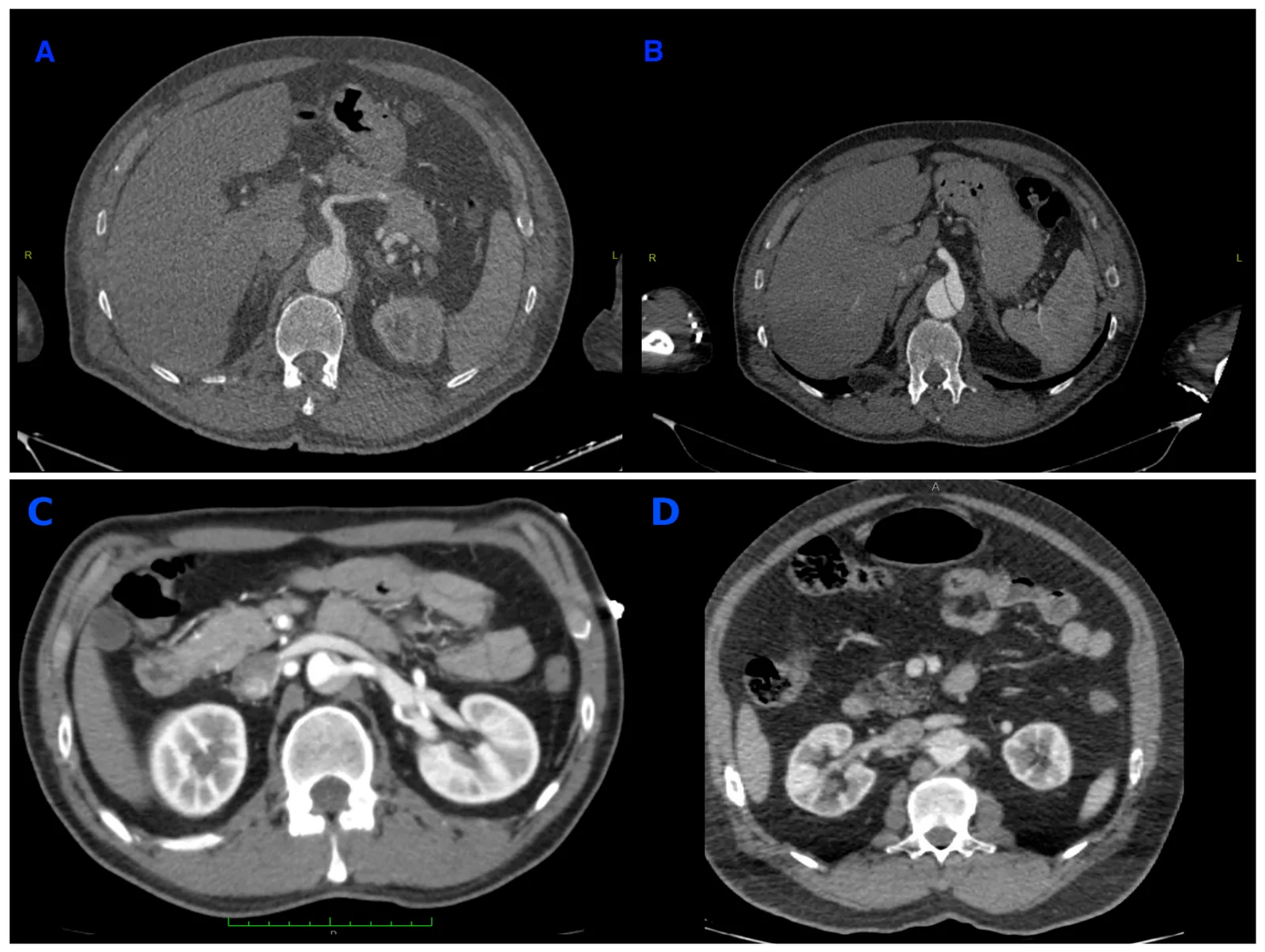
Branch-artery perfusion patterns on CT angiography. (**A**,**B**) In one patient, preoperative (**A**) true-lumen narrowing at the celiac level with predominant false-lumen perfusion; postoperative (**B**) expansion of the true-lumen area and increase in TLAR. (**C**) Dissection extending to the iliac arteries with both renal ostia intact and supplied from the true lumen. (**D**) Flap extending to both renal arteries with preserved balanced perfusion. R and L denote the patient’s right and left side (scanner laterality markers). TLAR: adjusted true lumen ratio.

Type 1 aortic dissection is therefore not merely an anatomical lesion but a time-critical systemic circulatory disorder. The flap propagates distally to involve the arch, visceral and iliac branches; owing to the position of the branch ostia, the presence of re-entry tears and the instantaneous effective flow, one branch may appear to be supplied from the true lumen while its neighbor is supplied from the false lumen. Branch opacification on CT is therefore not a fixed anatomical label but a dynamic indicator of functional perfusion (Figure 4) [11,16]. Central repair raises true-lumen pressure, but distal re-entry may prevent complete false-lumen collapse [16,22]; this is the anatomical basis of the malperfusion-to-syndrome gradient observed for every organ in our cohort.

### 4.2. Frequency of Malperfusion, Number of Organs and Organ-Specific Danger

The effect of malperfusion is consistent: mortality was 58% versus 8% in the early series of Apaydın et al. [23], 26.8% versus 13.6% in the STS analysis [9], and 18–36% in pooled analysis [24]; the dose–response with organ count runs from 12.6% to 43.4% in GERAADA [6] and from 12.5% to 70.0% in our cohort. Across registries, malperfusion prevalence ranges from 33.6% in GERAADA (*n* = 2137) [6] and 42.2% in ERTAAD (*n* = 3902) [25] to 27.7% in the US STS database (*n* = 9958, 27% operative mortality) [9]; 36.7% of patients had multi-organ involvement in the series of Wolfe et al. [26]; RERIC (*n* = 502) reported malperfusion syndrome in 20.5% with mortality of 34.7%/61.9%/85.7% by organ count [27]; and NORCAAD reported 33% mortality with cardiac and 31% with mesenteric/gastrointestinal malperfusion [28]. Malperfusion is not a binary state but a dose-dependent risk spectrum. Across organ systems, however, frequency and lethality are not parallel: Wolfe et al. [26] reported cerebral (21.3%) and peripheral (13.9%) malperfusion as the most frequent and mesenteric (4.2%) and spinal (2.1%) as the rarest, whereas the order of lethality is nearly the reverse.

**Coronary malperfusion.** According to the Neri classification, coronary involvement is divided into three types on the basis of operative findings: type A, intimal disruption confined to the ostium; type B, extension of the dissection into the coronary artery; type C, coronary disruption [29]. Kreibich et al. [30] reported successful ostial repair in 88% of type A, 63% of type B and 0% of type C lesions and recommended CABG as the primary option for type C. Ischemic ECG findings may delay diagnosis (approximately 24 h vs. 4 h) and expose patients to inappropriate antithrombotic therapy [31]. The right coronary artery is more often involved, but the mortality relationship is inverse: 15% with isolated right, 47% with left and 100% with bilateral involvement [32]. In our cohort, coronary malperfusion occurred in 14 patients (17.5%), above the 1.7–15% range in the literature [31,32]. Distal extension cannot by itself explain a proximal complication at the root, and several factors are more likely to account for this frequency. First, the direction of causality is more plausibly the reverse: an entry tear at the root feeds the false lumen with the highest possible pressure and flow, which both threatens retrograde rupture into the pericardium and drives the dissection distally, whereas a more distal tear leaves relatively preserved true-lumen flow that resists collapse and limits propagation. A cohort selected for iliac extension is therefore enriched in root-origin tears—in IRAD, a root-origin flap was significantly more frequent in patients with extensive (mesenteric) malperfusion [21]—and a root-origin flap is also the substrate for ostial involvement. This is not a rule, but it is a more coherent physiology than attributing a proximal complication to distal extent. Second, our definition relied on a troponin threshold (>150 ng/L) and/or intraoperative confirmation rather than on angiographic or operative classification alone; 18 patients (22.5%) presented in shock or under CPR, and in that setting troponin may rise from global hypoperfusion or resuscitation-related injury rather than from ostial dissection, so part of the excess may be a classification effect even though the criterion was verified to be independent of tamponade. Third, a referral effect specific to coronary involvement: a dissection with ostial involvement presents with ischemic electrocardiographic changes and a rising troponin, and in peripheral hospitals such patients are often managed as an acute coronary syndrome with serial troponin measurements—and in some cases antiplatelet loading—before the diagnosis is made and the patient is transferred to our center [31]. The resulting delay prolongs coronary ischemia, so that transferred patients reach us with more advanced myocardial injury and a higher troponin, which both increases the likelihood of meeting the troponin criterion and worsens the prognosis of those who do. Mortality was 28.6% with isolated malperfusion and 85.7% in the seven patients who developed the syndrome.

**Cerebral malperfusion.** In the IRAD analysis of 1873 patients, 4.7% presented with stroke and 2.9% with coma; mortality under medical management was 76.2% in the stroke group and 100% in the coma group, falling to 27.0% and 44.0% after surgical repair, with resolution of postoperative stroke and coma in 84.3% and 78.8% [33]. Tsukube et al. [34] achieved 14% hospital mortality and 86% full recovery of consciousness with surgery within 5 h in comatose patients (GCS < 11), versus 67% and 17% in the delayed group. Girdauskas et al. [35] found no mortality difference in their series of 29 patients, 52% of whom recovered without sequelae. In the systematic review of 363 patients by Wang et al. [36], postoperative mortality was 20.1% and neurological recovery 54.3%. In our cohort, cerebral malperfusion was present in 27 patients; only 2 presented with lateralizing signs (hemiplegia or gaze deviation with anisocoria, both fatal), and in the rest the picture was one of altered consciousness in the setting of shock/tamponade/arrest. A depressed GCS in this setting has two possible explanations—branch-vessel obstruction and global hypoxic–ischemic encephalopathy from low output or arrest—and the two frequently coexist. We addressed this in three ways: the definition was anchored primarily on CTA (25 of 27 patients had documented innominate/carotid dissection or thrombosis, and only 2 were classified on GCS alone); the GCS used was the last value before induction, after initial resuscitation; and the two patients taken to surgery under CPR are acknowledged as unclassifiable (Section 4.8). Where imaging is available, diffusion-weighted magnetic resonance imaging distinguishes territorial infarction from the diffuse cortical and basal-ganglia pattern of hypoxic injury, but it is rarely feasible before emergency surgery; in practice, a GCS that improves after central repair argues for a perfusion-dependent mechanism, whereas persistent coma after restoration of flow argues for hypoxic injury, which is why we regard the postoperative trajectory as the clinically decisive variable.

**Mesenteric malperfusion.** In the IRAD cohort of 1809 patients, incidence was 3.8%; mortality was 95.2% after medical treatment, 72.7% after endovascular treatment and 41.7% after surgical/hybrid treatment [21]. Kawahito et al. [37] showed in a Japanese series of 1026 patients that mortality rose from 4.8% to 30.0% as the number of organ systems increased. In the series of Girdauskas et al. [35], mortality in the visceral malperfusion subgroup (*n* = 8) was 75%.

**Renal malperfusion.** Reported in approximately 5% of studies and associated with 21% in-hospital mortality; although an independent predictor of operative mortality, it was not associated with long-term mortality at 8-year follow-up [24]. Pacini et al. [27] reported 2.8% incidence, 46.2% postoperative renal failure and 64.3% mortality (OR = 9.5) in their cohort. In type 1 dissection, the left renal artery frequently originates from the false lumen, but because of bilateral redundancy a unilateral false-lumen origin may remain clinically silent; this is the rationale for not using anatomical origin alone as a criterion in our renal definition.

**Limb malperfusion.** A relatively frequent (15–40%) but less severe complication of acute type A aortic dissection; isolated limb ischemia has not been shown to be an independent mortality risk factor [38]. In the series of 32 patients by Girdauskas et al. [35], standard proximal repair restored flow in all patients, and mortality (20%) did not differ from patients without malperfusion. Proximal (iliofemoral) involvement is associated with higher mortality than distal involvement [38,39].

**Spinal malperfusion.** The rarest (0.3–4.8%) but one of the most severe types; it is mostly dynamic, and central repair—with lumbar CSF drainage if needed—is usually sufficient [32]. Sandhu et al. [40] reported 17% complete and 65% partial recovery after central repair in 23 patients; in the pooled analysis of Chandiramani et al. [24], incidence was 1% and mortality 18%. In our cohort, spinal malperfusion syndrome occurred in a single patient (CSF drainage; survived).

Mesenteric malperfusion sits at the dangerous end of the spectrum: it carries the highest adjusted odds ratio in IRAD [1]; in STS data, mesenteric and coronary, but not cerebral, malperfusion were independent predictors [9]; and its resolution rate is 0% for intestinal versus 80% for renal/cerebral involvement [41]. Our cohort mirrors this picture; the malperfusion-to-syndrome gradient was directionally consistent for every organ (Table 3, Figure 2B), confirming Norton et al.’s [32] definition of syndrome as the late stage accompanied by tissue injury.

### 4.3. Turning the Light on the Mesentery: Why So Lethal, Why So Silent?

Three factors converge. The first is biological speed: of all ischemic beds, the bowel tolerates hypoperfusion least well, because loss of the mucosal barrier allows endotoxin to enter the portal circulation and because the splanchnic vascular bed responds to a fall in flow of more than three-quarters with autoregulatory vasoconstriction that further compromises the ischemic segment [11,12]. The second is the poverty of clinical signs: in IRAD, abdominal pain was absent in about two-fifths of patients with mesenteric malperfusion, while a fifth of those with abdominal pain had no malperfusion at all [21], and in intubated or obtunded patients, the abdominal examination loses its value altogether. The third is the absence of an organ-specific test: unlike troponin for the heart or creatinine for the kidney, no laboratory marker points to the bowel, and single-phase CT cannot always distinguish dynamic from static obstruction [12]. Mesenteric ischemia is therefore monitored indirectly through lactate: in the Michigan series, lactate ≥6 mmol/L was an independent risk factor for death from organ failure (OR = 13.5) [13], and serial measurement is a reliable indicator of ongoing malperfusion [14,15]. Yet lactate is not mesentery-specific; shock, CPR, bypass itself and impaired clearance all raise it. The “silence” of mesenteric malperfusion emerges from these three factors, and by the time it is diagnosed, it is often too late; the death of all nine patients who developed mesenteric syndrome in our cohort is the stark expression of this reality.

### 4.4. From a Pioneering Observation to Registries and Operating-Room Realities

The idea that mesenteric malperfusion should be treated as a distinct patient group is not new. Apaydın et al. [23], in an early series of 108 patients, found intestinal ischemia to be the strongest preoperative predictor of hospital mortality (RR = 39.4) and proposed that patients with severe visceral malperfusion be considered an exception to the policy of emergency ascending replacement—a pioneering observation made years before the modern “distal-first” literature; Yagdi et al. [42] reported visceral malperfusion as the strongest predictor of mortality (OR = 25.09). IRAD [10], founded in the same period, opened the way to systematic evaluation of malperfusion, and registries led by GERAADA [6] and ERTAAD [7], together with NORCAAD [19], UK-NACSA [8], RERIC [27], RESA-II [20], Sino-RAD [43] and JRAD [44], extended this to different populations; IRAD’s 17-year trend analysis documented a decline in mortality paralleling advances in surgical technique [4], and its 25-year experience an accumulation of 11,000 patients [5].

Two practical realities receive little emphasis in this literature; we state explicitly that they were not systematically measured in this cohort and derive from operating-room experience, so they are offered as observations to be tested rather than as results. The first is the site and timing of lactate sampling. Blood-gas samples are, by habit, arterial. With axillary cannulation the radial artery receives oxygenated pump blood, so an arterial sample reflects what the pump delivers rather than what the tissues produce, and lactate accumulating in an under-perfused lower body is diluted before it is measured; a sample drawn from the venous line entering the pump reflects the systemic burden more closely. The timing matters as much as the site, and it helps to follow the operation step by step. The patient arrives with a high lactate. Once bypass is established and the cross-clamp applied, the ascending entry tear lies proximal to the clamp and is excluded; the lower body is now perfused by pump flow from the axillary artery through the arch, a collapsed true lumen is partly re-expanded by that flow, and a second tear in the arch may still let pump flow enter the false lumen—so the lower body is perfused, but only partially and through dissected anatomy, and lactate may continue to rise while the root, valve or proximal anastomosis is being completed. During antegrade cerebral perfusion or circulatory arrest only the brain is perfused; the cooled lower body is not, lactate accumulates there unseen, and when the distal anastomosis is completed, false-lumen inflow excluded and full flow restored, the accumulated lactate is washed into the circulation and the value rises steeply—in our experience from 2–3 mmol/L to 15–20 mmol/L. This surge is expected (the washout of lactate from reperfused tissue) and is consistent with published data showing that the lactate rise across bypass, rather than any single value, carries prognostic information [14,15], and is not in itself a warning sign. What may be informative is the trend that follows, while the patient is still on bypass with full flow. Central repair restores true-lumen pressure and resolves dynamic obstruction in roughly four of five cases, whereas static obstruction—intra-branch dissection or thrombosis—resolves in only about a third [11,32]; this is the same reasoning behind the Michigan group’s critique of the TEVAR-first strategy, which corrects dynamic but not static malperfusion [32,45]. If, after the expected surge, venous lactate begins to fall as the lower body is reperfused, the obstruction was presumably dynamic and has resolved; if it fails to fall or keeps rising, a static component may be persisting, branch dissection may not resolve on postoperative CT, and—where mesenteric malperfusion was present preoperatively—the mesentery may still be the source. Read in source–clearance terms, the venous trend may thus indicate whether the lower-body source has been removed. The practical point concerns the unstable patient, in whom central repair cannot be deferred: a static mesenteric obstruction will not be corrected by the repair, and deferring the mesentery to the intensive care unit may mean arriving too late, yet a laparotomy, extended incision, bypass or fenestration in the same session should not be undertaken without reason. A venous lactate trend obtained while still on bypass may help to separate the patient who needs that step from the one who does not. This remains an observation to be tested, not an established rule. The second is the anatomical and ergonomic barrier: the mesenteric branches are retroperitoneal; opening a second cavity while bypass lines occupy the table, fear of bleeding in a heparinized patient and reluctance to prolong the case, combined with the expectation that “dynamic obstruction will resolve anyway,” push mesenteric assessment into second place. Mesenteric malperfusion is “insidious” in surgical workflow as well; when central repair is mandatory in an unstable patient, deferring the mesentery to the postoperative period becomes risky in light of the 6 h margin of Huang et al. [46].

### 4.5. Proximal-First or Distal-First?

The timing debate in mesenteric malperfusion arises from the coexistence of two opposing risks: delaying proximal repair carries the risk of rupture and tamponade, delaying mesenteric reperfusion carries the risk of irreversible bowel necrosis. The traditional “proximal-first” (central-repair-first) approach rests on the rationale that closure of the entry tear raises true-lumen pressure, resolves dynamic obstruction and eliminates rupture risk; Kawahito et al. [37] reported graded but acceptable outcomes by organ count with an emergency-central-repair policy, Yamamoto et al. [47] described concurrent direct mesenteric perfusion with central repair, and the Pennsylvania group [48] concurrent antegrade thoracic endovascular aortic repair (TEVAR). The systematic review of 352 patients by Wang et al. [49] is the strongest support for this camp: no mortality difference between central-repair-first (40.3%) and SMA-reperfusion-first (33.9%) strategies (*p* = 0.254), whereas endovascular-only (72.7%) and medical-only (93.1%) treatment fared markedly worse.

The Michigan group founded the “distal-first” (reperfusion-first) camp. Since 1996, Yang et al. [13] have reperfused patients with mesenteric malperfusion syndrome by endovascular fenestration/stenting before delayed aortic repair; over 20 years, in-hospital mortality fell from 21.0% to 10.7%, the median interval from intervention to open repair shortened from 7 to 4 days, and death from rupture was eliminated. Norton et al. [32] clarified the philosophical basis of this approach: dynamic obstruction can be medically corrected and treated by emergency repair, whereas static obstruction must be handled differently; when critical organ failure is present and the patient is stable, interventional radiology first is beneficial. The same group highlighted a critical distinction against the Emory TEVAR-first strategy [45]: TEVAR resolves only dynamic malperfusion, not static obstruction; indeed, 40% of patients in the TEVAR-first series required additional branch stenting. The distal-first paradigm has also been adopted by the Duke [12] and Melbourne [50] groups; Huang et al. [46] proposed a visceral-function-focused 6 h time threshold based on symptom duration. This debate is reflected in the guidelines: the 2022 American College of Cardiology/American Heart Association (ACC/AHA) [51] and 2024 ESC [52] guidelines strongly recommend central repair for malperfusion in general (class of recommendation, COR I) but assign a more flexible class (COR IIa) to mesenteric malperfusion; the AHA regards central repair and mesenteric revascularization as equivalent, and the ESC recommends invasive angiographic evaluation before or immediately after central repair. One point deserves emphasis: in the Michigan staged strategy, the patient is observed in the intensive care unit, not in a hybrid operating room, after endovascular reperfusion, and open repair is performed days later. Distal-first therefore requires a catheterization laboratory and interventional radiology expertise rather than a hybrid room; the same laboratory can be used for persistent malperfusion early after central repair. The real contribution of a hybrid room is the ability to combine both procedures in a single session under more controlled conditions—catheterization laboratories are not environments specialized for open surgery. The essence of what Yamamoto et al. [47] called the “fog of uncertainty” is that the right strategy depends on the patient, the obstruction mechanism and the resources of the center.

Right axillary cannulation, our standard, secures antegrade true-lumen flow. Direct aortic cannulation under epiaortic ultrasound guidance, or the Samurai cannulation described by Kitamura et al. [53], are alternatives that maintain true-lumen perfusion until definitive repair. Femoral cannulation, still widely used for ease of access, carries the risks of retrograde thrombus embolization, flap displacement to a worse position with unverified lumen cannulation, and rupture when run at high pressure; however, in patients with mesenteric malperfusion, transesophageal echocardiography (TEE)-verified true-lumen femoral cannulation may partially restore mesenteric supply by retrograde pressurization of the true lumen—Ito et al. [54] described exactly this strategy, monitoring SMA flow with intraoperative TEE.

### 4.6. The Source–Clearance Hypothesis and Our Supporting Findings

Hyperlactatemia does not arise from a single mechanism: (i) source—increased production by bowel ischemia, peripheral malperfusion or ischemia-reperfusion; (ii) clearance—failure to metabolize lactate because of impaired hepatic perfusion, renal function or oxygen delivery. SMA involvement relates to production; celiac involvement, hepatic/renal dysfunction, anemia and low output relate to clearance; hence, high lactate does not always signify ongoing mesenteric ischemia. The two mechanisms feed each other: acidosis impairs cardiac output, worsens hepatic/renal perfusion, and a “malperfusion–metabolic failure vicious circle” is established. The clinical importance is that the same anatomical finding may demand different management priorities: rapid distal reperfusion where production dominates; central repair, true-lumen restoration and hemodynamic optimization where clearance failure dominates. LAR reduces this balance to a single number; albumin is a reserve marker reflecting capillary leak, inflammation and reduced hepatic synthesis, and in a MIMIC-IV cohort of 919 patients with acute aortic dissection, Xu et al. [55] found LAR to be independently and dose-dependently associated with 30-, 90- and 365-day mortality.

Our findings support this framework in four ways. First, mesenteric malperfusion increased mortality six-fold in the multivariable model independently of shock, GCS and LAR. Second, LAR remained independent in the same model and discriminated better than lactate or albumin alone (Figure 3B). Third, LAR and SMA origin pattern were uncorrelated, and their combination raised the base model for predicting mesenteric syndrome—an endpoint independent of the definition—from 0.861 to 0.955 (optimism-corrected 0.847 to 0.909) (Figure 3A): metabolic burden and anatomical pattern carry non-overlapping information. Fourth, mortality was 75% in the eight patients with mixed (T/F) SMA origin, who did not differ from the rest of the cohort in age, GCS, creatinine or LAR; they were, however, more often hemodynamically unstable at presentation (shock or CPR in 50.0% vs. 19.4%), so part of the excess mortality in this subgroup is attributable to hemodynamic status rather than to the origin pattern itself, and with only eight patients the two cannot be separated statistically. Even so, the pattern was not accompanied by a higher metabolic burden (LAR) or worse renal function, which suggests that it marks an anatomical substrate—an ostium straddling both lumens, whose perfusion is highly sensitive to the pressure gradient between them—rather than simply a sicker patient. In current practice, distal-first is discussed only in the presence of documented thrombosis, and our findings suggest that a mixed origin pattern may also serve as a warning sign. This does not conflict with the conclusion of Fikani et al. [18] that patient-level anatomical features do not predict which branch will be involved: our question concerns not involvement but the relationship between the origin pattern of an involved branch and outcome. The mixed (T/F) SMA origin pattern may itself be heterogeneous. In the classification of Nagamine et al. [17], pattern 2a (compensated flow via intra-branch re-entry) may allow balanced perfusion and recovery after central repair, whereas pattern 2b (intra-branch dissection without re-entry) may carry higher risk and warrant consideration of a distal-first strategy. Ikeno et al. [22] reported that aortic true-lumen morphology measured at the level of the diaphragm (concave/convex) was associated with the mechanism of SMA obstruction (approximately 70–72% concordance); however, back-calculated from the authors’ figures, the specificity of convex morphology for a non-dynamic mechanism is only about 44%, aortic morphology at the diaphragm may not be a reliable proxy for the mechanism at the branch ostium, and generalizability to the celiac, renal or iliac branches is unknown. The area-based definition of TLAR reflects a methodological choice: because flow capacity is proportional to the fourth power of luminal radius (Poiseuille), cross-sectional area may reflect flow capacity more directly than circumferential arc length [56]; in irregular (crescent-shaped) lumen geometries, however, even area may not fully capture hydraulic resistance. Phantom models have shown that true-lumen collapse varies with entry-tear size, false-lumen outflow resistance and cardiac output [57]. The finding is hypothesis-generating and requires prospective validation. The correlation of D-dimer with lactate/LAR and its inverse relationship with platelets suggest that consumptive coagulopathy progresses alongside metabolic burden and, combined with heparinization, may increase bleeding risk in the ischemic mesentery.

### 4.7. A Decision Pathway for Centers Without a Hybrid Operating Room

**Figure 5:**
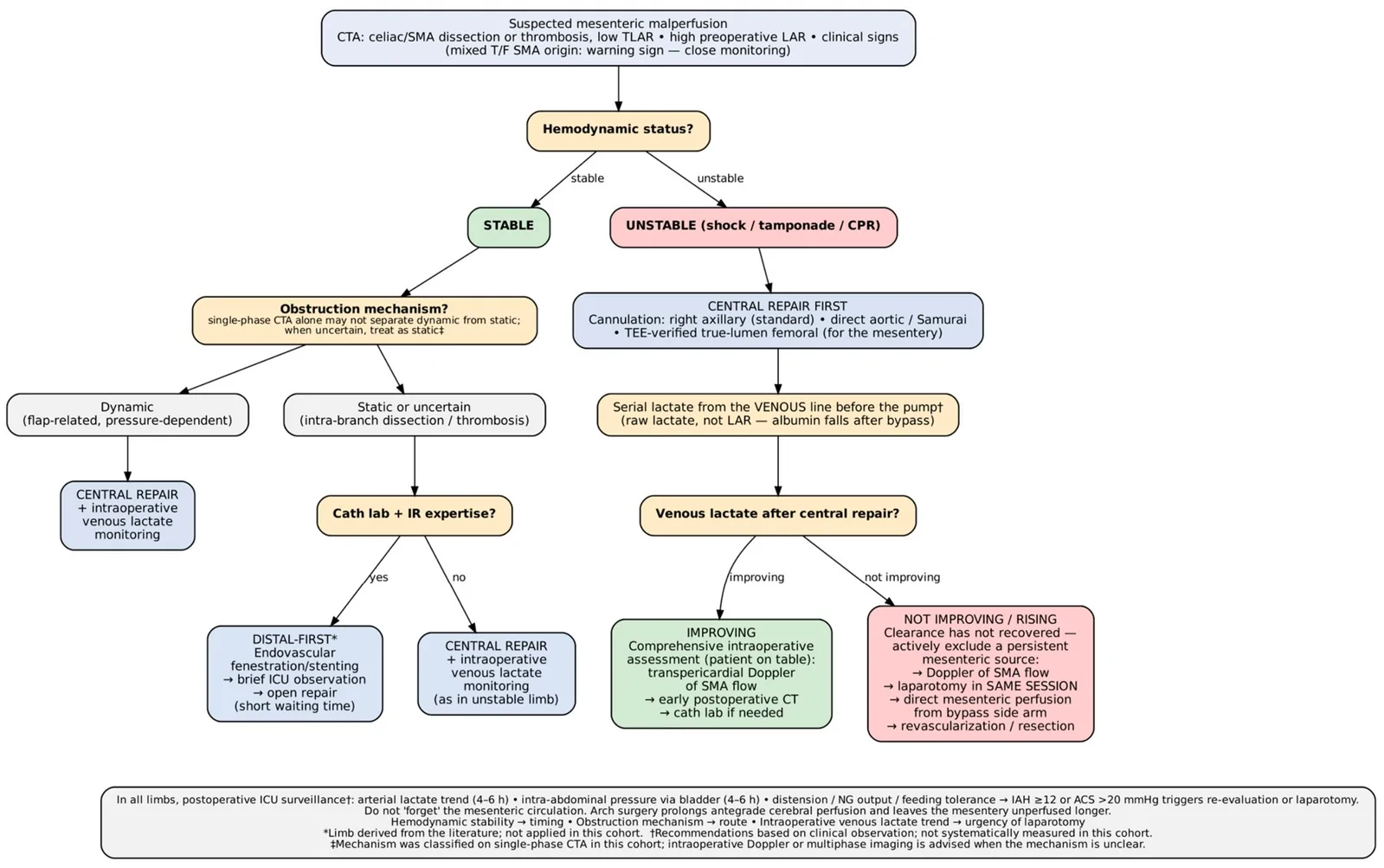
Decision pathway for mesenteric malperfusion in centers without a hybrid operating room. Mixed (T/F) SMA origin is a warning sign requiring close monitoring, not a decision criterion. Arrows indicate the sequence of decisions and actions; the label on an arrow states the condition that selects that branch, and arrows within a box (→) denote consecutive steps within the same branch. * The distal-first limb is derived from the literature and was not applied in this cohort. † Venous lactate monitoring and the postoperative surveillance bundle (lactate trend, intra-abdominal pressure, clinical abdominal assessment) are recommendations based on clinical observation and were not systematically measured in this cohort. ‡ The obstruction mechanism was classified on single-phase CTA in this cohort; when the mechanism is unclear, it should be treated as static and clarified by multiphase imaging or intraoperative Doppler. IR: interventional radiology; ICU: intensive care unit; TLAR: adjusted true lumen ratio; TEE: transesophageal echocardiography; NG: nasogastric; IAH: intra-abdominal hypertension; ACS: abdominal compartment syndrome.

In light of these findings, we propose a pragmatic decision pathway (Figure 5). The first step is hemodynamic status. **If the patient is stable**, the mechanism governs: with dynamic obstruction, central repair is usually sufficient; with static obstruction and access to a catheterization laboratory and interventional radiology expertise, endovascular fenestration/stenting first, brief intensive-care observation and open repair may be considered as in the Michigan model [13,32], keeping the waiting time short [13,46]. This limb depends on correct identification of the mechanism, and here the imaging basis of our study is a real constraint: single-phase CTA captures one instant of the cardiac cycle, so a flap that intermittently occludes the SMA ostium may appear patent, and a compressed but patent ostium may be indistinguishable from an early intra-branch dissection. In our cohort, static obstruction was therefore recognized only when intra-branch dissection or thrombosis was visible, and some dynamic lesions may have been under-called. For the pathway this has two practical consequences. First, the mechanism should be regarded as uncertain unless intra-branch dissection or thrombosis is seen, and an uncertain mechanism in a stable patient should be managed as static—that is, it should prompt additional assessment (electrocardiogram-gated or multiphase CTA, or intraoperative transpericardial/transesophageal Doppler of the SMA [54,58]) rather than an assumption that central repair will suffice. Second, the unstable limb of the pathway was deliberately designed not to depend on the mechanism: the venous lactate trend after central repair and intraoperative Doppler of SMA flow are functional checks that reveal whether the obstruction has resolved, whatever its mechanism. Mixed SMA origin is not a decision criterion but a warning sign requiring close monitoring. The distal-first limb was not applied in our cohort and is derived from the literature. **If the patient is unstable**, central aortic repair takes priority. Right axillary cannulation secures antegrade true-lumen flow, with direct aortic cannulation as an alternative; femoral cannulation risks retrograde embolization and wrong-lumen cannulation, although TEE-verified true-lumen femoral cannulation may partially restore mesenteric supply by retrograde pressurization [54]. Whichever cannulation is chosen, we recommend serial lactate sampling from the venous line entering the pump during and after central repair; this recommendation rests on physiological rationale and clinical observation rather than cohort data. Raw lactate, not LAR, should be monitored, because post-bypass hemodilution lowers albumin to 22–24 g/L and artificially raises LAR. If venous lactate does not improve after central repair, the source–clearance balance has not been restored: either production is continuing or clearance has not recovered, and the two cannot be separated from the lactate value alone—although, because full-flow bypass itself partly substitutes for clearance, a persistent rise under these conditions is harder to attribute to clearance failure alone. Because an unresolved mesenteric source is the most lethal and the most correctable of these possibilities, it must be actively excluded while the patient is still on the table—laparotomy in the same session, Doppler assessment of the SMA and, if needed, direct mesenteric perfusion from the bypass circuit [47], embolectomy/septectomy, bypass or resection should be performed—none requires endovascular equipment. If lactate improves, persistent malperfusion is assessed while the patient is still in the operating room as in the model of Abdellah et al. [59]; the approach of the Neri group, assessing SMA flow by transpericardial Doppler without laparotomy [58]—9.1% mortality when flow normalized versus 60% with persistent malperfusion—is a practical tool for this step. Early postoperative CT then follows. Postoperative ICU surveillance deserves the same structure, because mesenteric malperfusion may also declare itself only after central repair. We suggest a simple bundle that does not depend on imaging: the arterial lactate trend at 4–6 h intervals rather than any single value; serial intra-abdominal pressure measurement via bladder pressure at the same intervals; and a daily check of abdominal distension, nasogastric output and tolerance of early enteral feeding. Abdominal distension, whether from bowel edema after reperfusion or from evolving ischemia, raises intra-abdominal pressure and further reduces splanchnic perfusion pressure, so intra-abdominal hypertension (≥12 mmHg) or abdominal compartment syndrome (>20 mmHg with new organ dysfunction) [60] provides an objective trigger for re-evaluation—bedside ultrasound or CT where feasible, and decompressive or exploratory laparotomy where it is not. Like venous lactate sampling, this bundle is a recommendation derived from clinical reasoning and was not systematically applied in this cohort. In summary, hemodynamic stability determines timing, obstruction mechanism the route, and the intraoperative venous lactate trend the urgency of laparotomy; the postoperative lactate trend and intra-abdominal pressure the need for re-exploration; the key element is not technology but a culture of systematic perioperative vigilance.

Preoperative assessment also has a structural limit: the subgroup with normal preoperative GCS who deteriorated postoperatively (*n* = 18; mortality 77.8%) was characterized by prolonged pump times; no preoperative parameter can predict intraoperative events, and the death of all four FET patients underscores the importance of individualizing the extent of surgery [32]. The shorter CT-to-bypass time in non-survivors reflects the fastest transfer of the most unstable patients to the operating room.

### 4.8. Limitations

The single-center retrospective design is prone to selection bias. The sample size (*n* = 80; 27 events) produces wide confidence intervals in the multivariable model, and several subgroups on which key observations rest are small—mesenteric syndrome (*n* = 9), mixed SMA origin (*n* = 8), involvement of three or more organs (*n* = 6) and postoperative ECMO (*n* = 4). With these numbers, the statistical power to detect or exclude moderate effects is low, point estimates such as the 75% mortality with mixed SMA origin or the 100% mortality with mesenteric syndrome carry wide uncertainty (for 9/9, the 95% CI extends down to 66%), and the incremental AUC for mesenteric syndrome, although preserved on bootstrap validation, was derived from nine events; all subgroup findings are therefore hypothesis-generating and their generalizability is limited. Because the mesenteric definition incorporates TLAR and lactate, discrimination was analyzed against mesenteric syndrome, an endpoint independent of the definition. Branch patterns rely on single-phase CT, which cannot show flap motion; the dynamic/static classification is therefore approximate, dynamic obstruction may have been under-recognized, and TLAR may vary with cardiac phase in dynamic obstruction; in addition, measurements were performed by a single observer, so inter-observer reproducibility was not assessed. The LAR protocol used a single preoperative arterial sample; serial preoperative values and venous-line lactate during bypass were not recorded, so the intraoperative lactate recommendation in Section 4.7 is based on clinical observation rather than on data from this cohort. The iliac-extension criterion limits generalizability, and 64 of 144 patients with type I dissection (44%) could not be analyzed because preoperative CTA was not available for review; unavailability was related to the source of imaging and to record-access rules rather than to clinical status, and 30-day mortality did not differ significantly between excluded and included patients (48.4% vs. 33.8%; *p* = 0.09), although the trend toward higher mortality among excluded patients means that the cohort may underestimate overall institutional mortality and selection bias cannot be entirely excluded. Postoperative missing data are not random (MNAR); the primary analysis, based entirely on complete preoperative variables, is unaffected, but AKI and syndrome analyses should be interpreted with caution. The cerebral malperfusion definition combined an anatomical criterion with GCS; although only 2 of 27 patients were classified on GCS alone, in patients presenting in shock or under CPR, a depressed GCS cannot be attributed with certainty to branch-vessel obstruction rather than to global hypoxic–ischemic injury, and preoperative neuroimaging beyond CTA was not performed systematically: in a few patients the dissection was in fact discovered incidentally on brain CT or MRI obtained for altered consciousness, but such studies were not available for all patients and were not analyzed. Comparison with existing risk scores was beyond the scope of this study. Finally, the source–clearance model and the decision pathway are a conceptual framework; venous lactate monitoring and postoperative intra-abdominal pressure surveillance are recommendations, the distal-first limb was not applied in this cohort, and the framework has not been tested prospectively.

## 5. Conclusions

In acute type 1 aortic dissection with iliac extension, malperfusion is a dose-dependent risk spectrum that escalates with the number of organs involved and with progression to syndrome. Mesenteric malperfusion is the most lethal component, increasing mortality six-fold independently of shock, GCS and LAR; none of the nine patients in our series who progressed to mesenteric syndrome survived, an observation that, although drawn from a small subgroup, underscores the need for early recognition. In cerebral malperfusion, prognosis is determined by the postoperative neurological course. Preoperative LAR and SMA origin pattern carry independent and complementary information on mesenteric risk; mixed SMA origin may, at a hypothesis-generating level, identify a high-risk subgroup not captured by routine criteria. The source–clearance framework and the proposed decision pathway offer a validation-pending but practicable approach to ensuring that the mesenteric circulation is “not forgotten” in centers without a hybrid operating room.

## Figures and Tables

**Table 1:** Selected baseline, laboratory and malperfusion findings by 30-day mortality.

Variable	Survivors (*n* = 53)	Non-Survivors (*n* = 27)	*p*
Age (years), mean ± SD	54.7 ± 11.8	59.7 ± 12.4	0.08
Preoperative GCS, median [IQR]	15 [15–15]	15 [7.5–15]	0.003
Tamponade, *n* (%)	3 (5.7)	8 (29.6)	0.01
Hemodynamics (stable/shock/CPR), *n*	48/5/0	14/11/2	0.01
CT-to-bypass time (min), median [IQR]	241 [178–326]	192 [166–230]	0.034
Platelets (×10^3^/µL), mean ± SD	200.8 ± 59.3	168.8 ± 48.8	0.01
Albumin (g/L), mean ± SD	35.7 ± 7.0	30.5 ± 9.0	0.01
Creatinine (mg/dL), mean ± SD	1.08 ± 0.33	1.46 ± 0.81	0.03
Lactate (mmol/L), median [IQR]	2.1 [1.2–2.9]	3.4 [2.6–6.8]	0.002
LAR, median [IQR]	0.059 [0.030–0.086]	0.118 [0.072–0.233]	<0.001
Lactate after bypass (mmol/L), mean ± SD	7.3 ± 4.3	14.3 ± 5.9	<0.001
Cerebral malperfusion, *n* (%)	11 (20.8)	16 (59.3)	0.001
Mesenteric malperfusion, *n* (%)	6 (11.3)	15 (55.6)	<0.001
Coronary malperfusion, *n* (%)	6 (11.3)	8 (29.6)	0.06
Renal malperfusion, *n* (%)	7 (13.2)	3 (11.1)	1.00
Iliac malperfusion, *n* (%)	4 (7.5)	3 (11.1)	0.68
SMA origin (T/F/T-F), *n*	43/8/2	16/5/6	0.03
Celiac origin (T/F/T-F), *n*	39/11/3	15/9/3	0.26

Continuous variables are compared with the t-test (mean ± SD) or Mann–Whitney U test (median [IQR]); categorical variables with the chi-square or Fisher’s exact test. No differences were found in sex, BMI, comorbidities, EF, aortic regurgitation, WBC, hemoglobin, CRP, preoperative troponin, ALT/AST, D-dimer, renal origins, TLR values, arch dissection or iliac thrombosis (Supplementary Tables S1 and S2). Spinal malperfusion occurred in 1 patient. GCS: Glasgow Coma Scale; CPR: cardiopulmonary resuscitation; LAR: lactate-to-albumin ratio; SMA: superior mesenteric artery; T: true lumen; F: false lumen; T-F: mixed origin.

**Table 2:** Multivariable logistic regression for 30-day mortality.

Variable	B (SE)	*p*	OR (95% CI)
Preoperative shock/CPR	1.949 (0.751)	0.010	7.02 (1.61–30.60)
Mesenteric malperfusion	1.830 (0.733)	0.013	6.24 (1.48–26.22)
Preoperative LAR (per 0.01 unit)	0.070 (0.031)	0.026	1.07 (1.01–1.14)
Preoperative GCS (per point)	−0.289 (0.127)	0.023	0.75 (0.58–0.96)

Model χ^2^(4) = 41.76; *p* < 0.001; Nagelkerke R^2^ = 0.564; Hosmer–Lemeshow *p* = 0.61; apparent AUC = 0.894 (bootstrap percentile 95% CI 0.814–0.967); optimism-corrected AUC = 0.876. Tamponade and thrombocytopenia lost significance in the full model (*p* = 0.32 and 0.36); tamponade was removed as collinear with shock; cerebral malperfusion was not entered separately because of its overlap with GCS (r = −0.62). B: regression coefficient; SE: standard error; OR: odds ratio; CI: confidence interval.

**Table 3:** Organ-specific malperfusion, malperfusion syndrome and 30-day mortality.

Organ	Malperfusion *n* (%)	Mortality % (Deaths/*n*)	OR	*p*	Mortality Without Syndrome	Mortality with Syndrome
Mesenteric	21 (26.2)	71.4 (15/21)	9.79	<0.001	6/12 (50.0)	9/9 (100)
Cerebral	27 (33.8)	59.3 (16/27)	5.38	0.001	0/9 (0)	16/18 (88.9)
Coronary	14 (17.5)	57.1 (8/14)	3.30	0.06	2/7 (28.6)	6/7 (85.7)
Iliac	7 (8.8)	42.9 (3/7)	1.53	0.68	0/4 (0)	3/3 (100)
Renal	10 (12.5)	30.0 (3/10)	0.82	1.00	1/7 (14.3)	2/3 (66.7)
**Number of organs involved**						
0	32 (40.0)	12.5 (4/32)				
1	28 (35.0)	32.1 (9/28)				
2	14 (17.5)	71.4 (10/14)				
≥3	6 (7.5)	66.7 (4/6)				

Syndrome columns show the distribution among patients with a preoperative malperfusion finding; patients who developed a syndrome without a preoperative finding (cerebral 4, coronary 3, renal 2) are not included. OR and *p* (Fisher’s exact test) compare patients with versus without malperfusion of each organ; χ^2^ = 21.27, df = 4, *p* < 0.001 for mortality across number of organs involved. The ≥3-organ group comprises only 6 patients; its point estimate is imprecise.

## Data Availability

The de-identified dataset supporting the findings is available from the corresponding author on reasonable request.

## Supplementary Materials

The following supporting information can be downloaded at: https://www.mdpi.com/article/10.3390/jcm15207958/s1, Table S1: Baseline and laboratory variables by 30-day mortality (full list); Table S2: Radiological and operative variables by 30-day mortality; Table S3: Univariable logistic regression for 30-day mortality.

## Abbreviations

ACC/AHA: American College of Cardiology/American Heart Association; AKI: acute kidney injury; AUC: area under the receiver operating characteristic curve; BMI: body mass index; CABG: coronary artery bypass grafting; CI: confidence interval; COR: class of recommendation; CPR: cardiopulmonary resuscitation; CRP: C-reactive protein; CSF: cerebrospinal fluid; CT(A): computed tomography (angiography); ECMO: extracorporeal membrane oxygenation; ERTAAD: European Registry of Type A Aortic Dissection; ESC: European Society of Cardiology; FET: frozen elephant trunk; GCS: Glasgow Coma Scale; GERAADA: German Registry for Acute Aortic Dissection Type A; ICU: intensive care unit; IQR: interquartile range; IRAD: International Registry of Acute Aortic Dissection; KDIGO: Kidney Disease: Improving Global Outcomes; LAR: lactate-to-albumin ratio; MNAR: missing not at random; OR: odds ratio; PCI: percutaneous coronary intervention; ROC: receiver operating characteristic; SD: standard deviation; SMA: superior mesenteric artery; STS: Society of Thoracic Surgeons; TEE: transesophageal echocardiography; TEVAR: thoracic endovascular aortic repair; TLAR: adjusted true lumen ratio; TLR: true lumen ratio.
