# Supplementary material for: Organ Malperfusion and Mortality in Acute Type 1 Aortic Dissection with Iliac Extension: A Retrospective Single-Center Study Focused on Mesenteric Malperfusion and the Source–Clearance Hypothesis
Source: J Clin Med. 2026 Oct 14;15(20):7958. doi: 10.3390/jcm15207958 (PMC13705978; doi:10.3390/jcm15207958)
Supplement: Supplementary file 1 [file jcm-15-07958-s001.zip › jcm-4622230-supplementary.pdf]

SUPPLEMENTARY MATERIAL

Organ Malperfusion and Mortality in Acute Type 1 Aortic Dissection with Iliac Extension: A Retrospective Single-Center Study Focused on Mesenteric Malperfusion and the Source–Clearance Hypothesis

Ali Önder Kılıç

Supplementary Tables

Supplementary Table S1. Baseline characteristics and laboratory findings by mortality (full list)

Variable	Survivors (n=53)	Non-survivors (n=27)	p
Age (years)	54.7±11.8	59.7±12.4	0.08
Male sex, n (%)	37 (69.8)	24 (88.9)	0.06
Preoperative GCS, median [IQR]	15 [15–15]	15 [7.5–15]	0.003
Postoperative GCS, median [IQR]	15 [15–15]	3 [3–3]	<0.001
Tamponade, n (%)	3 (5.7)	8 (29.6)	0.01
Hemodynamics (stable/shock/CPR)	48/5/0	14/11/2	0.01
CT-to-bypass time (min), median [IQR]	241 [178–326]	192 [166–230]	0.034
**Preoperative laboratory**			
Platelets (×10³/µL), mean±SD	200.8±59.3	168.8±48.8	0.01
Albumin (g/L), mean±SD	35.7±7.0	30.5±9.0	0.01
Creatinine (mg/dL), mean±SD	1.08±0.33	1.46±0.81	0.03
Lactate (mmol/L), median [IQR]	2.1 [1.2–2.9]	3.4 [2.6–6.8]	0.002
LAR, median [IQR]	0.059 [0.030–0.086]	0.118 [0.072–0.233]	<0.001
D-dimer	7.3	8.4	0.09
**Postoperative laboratory**			
Lactate, post-bypass (mmol/L), mean±SD	7.3±4.3	14.3±5.9	<0.001
Lactate, ICU (mmol/L), median [IQR]	4.8 [2.5–8.0]	17.1 [8.0–20.8]	<0.001
LAR, post-bypass, median [IQR]	0.203 [0.116–0.383]	0.600 [0.339–0.927]	<0.001
Creatinine, postoperative (mg/dL), median [IQR]	1.45 [1.05–1.90]	2.33 [1.78–3.69]	0.002
AKI stage (mean)	1.0	1.9	0.002

No differences in BMI, diabetes, hypertension, COPD, EF, aortic regurgitation grade, WBC, hemoglobin, hematocrit, CRP, preoperative troponin, ALT or AST (p>0.05). Postoperative variables are based on complete-case analysis.

Supplementary Table S2. Malperfusion and anatomical/radiological findings by mortality (full list)

Variable	Survivors (n=53)	Non-survivors (n=27)	p

Variable	Survivors (n=53)	Non-survivors (n=27)	p
**Malperfusion, n (%)**			
Cerebral	11 (20.8)	16 (59.3)	0.001
Mesenteric	6 (11.3)	15 (55.6)	0.0001
Coronary	6 (11.3)	8 (29.6)	0.06
Renal	7 (13.2)	3 (11.1)	1.00
Iliac	4 (7.5)	3 (11.1)	0.68
**Branch origin (T/F/T-F)**			
SMA	43/8/2	16/5/6	0.03
Celiac	39/11/3	15/9/3	0.26
Right renal	34/16/3	23/4/0	0.11
Left renal	23/25/5	10/17/0	0.17
**TLR (mean %)**			
Celiac	44.4	47.2	0.88
SMA	45.5	41.7	0.40
Right renal	51.1	45.8	0.35
Left renal	55.8	66.2	0.04

Spinal malperfusion occurred in 1 patient (CSF drainage; survived). Arch dissection and iliac thrombosis were not associated with mortality.

Supplementary Table S3. Univariable logistic regression of mortality risk factors

Variable	OR	95% CI	p
Preoperative shock/CPR	8.91	2.71–29.33	0.0003
Mesenteric malperfusion	9.79	3.13–30.60	0.0001
Tamponade	7.02	1.68–29.27	0.008
Cerebral malperfusion	5.55	2.01–15.32	0.001
Preoperative creatinine (mg/dL)	5.17	1.42–18.78	0.013
Thrombocytopenia (<150×10³/µL)	3.36	1.18–9.61	0.024
Coronary malperfusion	3.30	1.01–10.79	0.048
Preoperative lactate (mmol/L)	1.35	1.10–1.64	0.004
Preoperative LAR (per 0.01 unit)	1.09	1.03–1.15	0.004
Preoperative GCS (per point)	0.70	0.56–0.87	0.002
Age (years)	1.04	0.995–1.08	0.09
Left renal TLR (%)	1.02	0.999–1.04	0.06

Renal and iliac malperfusion, BMI, sex, comorbidities, and celiac/SMA/right renal TLR were not associated with mortality.
